# Conserved positive selection signals in gp41 across multiple subtypes and difference in selection signals detectable in gp41 sequences sampled during acute and chronic HIV-1 subtype C infection

**DOI:** 10.1186/1743-422X-5-141

**Published:** 2008-11-24

**Authors:** Gama P Bandawe, Darren P Martin, Florette Treurnicht, Koleka Mlisana, Salim S Abdool Karim, Carolyn Williamson, The CAPRISA 002 Acute Infection Study Team

**Affiliations:** 1 Institute of Infectious Disease and Molecular Medicine, Faculty of Health Sciences, University of Cape Town, Anzio Road, Observatory, 7925, South Africa; 2 Doris Duke Medical Research Institute, Nelson R Mandela School of Medicine, University of KwaZulu-Natal, Private Bag X7, Congella, 4013, South Africa

## Abstract

**Background:**

The high diversity of HIV variants driving the global AIDS epidemic has caused many to doubt whether an effective vaccine against the virus is possible. However, by identifying the selective forces that are driving the ongoing diversification of HIV and characterising their genetic consequences, it may be possible to design vaccines that pre-empt some of the virus' more common evasion tactics. One component of such vaccines might be the envelope protein, gp41. Besides being targeted by both the humoral and cellular arms of the immune system this protein mediates fusion between viral and target cell membranes and is likely to be a primary determinant of HIV transmissibility.

**Results:**

Using recombination aware analysis tools we compared site specific signals of selection in gp41 sequences from different HIV-1 M subtypes and circulating recombinant forms and identified twelve sites evolving under positive selection across multiple major HIV-1 lineages. To identify evidence of selection operating during transmission our analysis included two matched datasets sampled from patients with acute or chronic subtype C infections. We identified six gp41 sites apparently evolving under different selection pressures during acute and chronic HIV-1 infections. These sites mostly fell within functional gp41 domains, with one site located within the epitope recognised by the broadly neutralizing antibody, 4E10.

**Conclusion:**

Whereas these six sites are potentially determinants of fitness and are therefore good candidate targets for subtype-C specific vaccines, the twelve sites evolving under diversifying selection across multiple subtypes might make good candidate targets for broadly protective vaccines.

## Background

Detailed characterisation of the selective forces that are shaping HIV-1 evolution is crucial if we are to fundamentally understand HIV pathogenesis. To design vaccines that will protect against HIV, we might ultimately require accurate predictive models of how particular viral proteins will evolve in response to particular selection pressures.

To avoid host immune responses, the virus' survival strategy is dominated by high mutation and recombination rates that, while possibly jeopardizing its long term survival as a species, guarantees its short term success [1]. This selection for continual change, called positive (or diversifying) selection, is driving HIV evolution against a background of negative (or purifying) selection favouring preservation of functionally important protein sequences [2]. Thus, HIV evolution is characterised by a perpetual tug-of-war between the immediate short term benefits of positively selected immune escape mutations, and the long term selective advantages of maintaining optimal protein function [3,4].

These conflicting forces are perhaps most manifest within the *env *gene that encodes the HIV envelope proteins. The HIV envelope is made up of two components: gp120 and gp41. These two proteins are targeted by both the humoral and cellular arms of the immune system. Whereas positive selection that is detectable in parts of *env *encoding the exposed surfaces of gp120 is most likely driven by the need for the virus to escape either neutralizing antibodies [5,6] or cytotoxic T lymphocytes, positive selection at sites encoding unexposed residues is presumably driven by selection for both escape from cytotoxic T lymphocytes and altered cell tropism [7-13]. Although certain regions of *env *are particularly accommodating of positive selection, most codons are functionally important and as a consequence many residues are detectably evolving under negative selection [14].

Both gp120 and gp41 have functionally distinct but additive roles in HIV infection and pathogenesis [15]. While gp120 mediates entry via CD4 and co-receptor binding, gp41 is essential for post receptor binding events including viral fusion and assembly [16-20]. Despite these gp41 mediated processes being amongst the most significant determinants of replicative capacity and pathogenic potential in any given strain [21] there has been much more research focused on the selective forces acting on its partner, gp120.

Recently emphasis has been placed on the study of viruses sampled close to transmission (during acute and early infection) based largely on the premise that protection against these variants must be the primary target of vaccine and microbicide development strategies. HIV is believed to experience extremely severe population bottlenecks during transmission with usually only one, or at most a few, genetic variants establishing an infection within a new host [14,22,23]. As a large proportion of transmissions are thought to occur during the acute phase of infection [24], evolutionary innovations arising early on in infections may also be disproportionately important for the long-term evolution of HIV in that many selectively advantageous mutations occurring later in infections have a greater chance of "missing the boat" for transmission [25]. The viruses that make it through the transmission bottleneck may contain a lot of immune evasion mutations that are irrelevant or possibly even evolutionarily harmful within the context of their new host's immune environment. It would be expected that many of these formerly useful mutations – especially those with associated replicative fitness costs – would be strongly selected against [26-28]. While the evolutionary relevance of "transmission fitness" and the "transmission sieve" in HIV [29,30] are currently under debate (see Lemey *et al *[31] for a review), it is widely acknowledged that the reversion of immune escape mutations that incur replicative fitness costs is a prominent feature of HIV evolution [27,32,33].

Given that (i) transmission may selectively favour genotypes with high transmission fitness, (ii) recently transmitted viruses will have, on average, spent a greater proportion of their evolutionary histories in acute infections than viruses sampled during chronic infections and (iii) transmitted viruses generally enter an environment selectively favouring the rapid reversion of some former immune evasion mutations, we anticipated that the genes of recently transmitted viruses might display marks of selection that differentiated them from viruses sampled during chronic infections.

We show here that whereas signals of selection in gp41 are largely conserved between both different HIV subtypes and viruses sampled during different stages of HIV infections, at least six sites in gp41 display signals of selection that appear to differentiate viruses sampled during acute and chronic infections.

## Results

### Recombination in gp41

**Figure 1:**
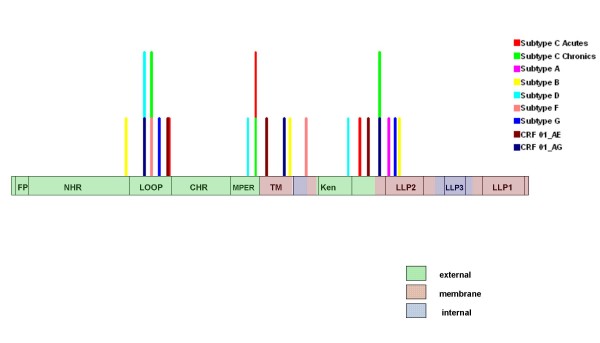
Distribution of recombination breakpoints across the gp41 encoding region of two subtype C datasets and seven other subtypes/circulating recombinant forms as detected by the GARD method. The positions at which recombination breakpoints are inferred to have occurred in the different datasets are illustrated using vertical coloured lines specific for each dataset.

As recombination occurs at high frequencies during HIV infections [34-36] and can seriously confound inferences of positive selection [37-39] it was necessary to account for the positions of recombination breakpoints in nine gp41 datasets drawn from different subtypes and circulating recombinant forms. The presence of potential recombination breakpoints in these datasets was first determined using the GARD method [40]. The distribution of detected breakpoints was apparently non-random with three breakpoint clusters identified (Figure 1): one in the loop region; the second around the major trans-membrane domain; and the third in the region downstream of the Kennedy sequence into the LLP2 domain. Analysis using alternative recombination analysis methods implemented in the program RDP3 [41] confirmed that breakpoints clustering around the transmembrane domain constituted evidence of a statistically significant (global P < 0.01) recombination hotspot (Additional file 1). This result supports a recent claim that gp41 is the site of a major "inter-subtype" recombination hotspot in HIV-1M genomes [42]. In fact the breakpoint hotspot detected in the part of gp41 encoding the transmembrane domain maps to almost precisely the location identified by Fan *et al *[43].

None of the three areas of gp41 where breakpoint clusters were observed contain predicted hairpins or other detectable RNA-secondary structures that might have mechanistically predisposed these regions to recombination. Besides being caused by biochemical predispositions to recombination, recombination hotspots are also potentially caused by purifying selection acting on defective recombinants. By culling recombinants that are less viable than parental viruses, purifying selection will yield genomes with breakpoints clustered within genome regions that tolerate recombination well [44]. As with mutation events, it is probably most accurate to think of there being a continuum of different kinds of recombination events: From those that are lethal through those that are only mildly deleterious or neutral to those that are advantageous. Since the least deleterious recombination events tend to be those that exchange self-contained sequence "modules" which continue to function properly within the context of genomic backgrounds very different from those in which they evolved [45-47], it is possible that the recombination breakpoint clusters that are detectable in gp41 simply demarcate the main modules of this protein.

### Consistently detectable positive selection signals across multiple subtypes

Recombination breakpoints detected by GARD were taken into consideration during subsequent selection analyses. In order to get a comprehensive picture of selective forces acting on gp41 during HIV infections in general we examined the nine gp41 datasets using the SLAC, FEL and IFEL methods implemented in Hyphy. Although selection signals detectable in multiple HIV subtypes have already been described within gp41 [48,49], these signals were detected without taking recombination into account. Using the three recombination-aware selection analysis methods in Hyphy we collectively detected a total of 346 positive selection signals across all 9 datasets (59 by SLAC, 159 by FEL and 128 by IFEL) at 89 different sites within gp41. Purifying selection in gp41 is pervasive with 214 out of its 352 sites detectably evolving under purifying selection in at least one of the nine datasets.

Examination of every site that is detectably evolving under any form of selection in any of the datasets indicated varying levels of selection acting on the various gp41 domains. Analysing the ratio of sites evolving under positive and purifying selection in different parts of gp41 indicated that the LLP1 domain has the highest (0.578947) followed by the MPER (0.545455) and the loop region (0.461538). The fusion protein also has a high ratio of sites evolving under positive selection (0.428571). The trans-membrane domain (0.363636) and the C and N-heptad repeats (0.242424 and 0.184211, respectively) have the lowest ratios of positively:negatively selected sites. The trans-membrane domain is conserved and shares common characteristics with other viral and cellular membrane spanning domains [50-52] and is therefore unlikely to tolerate high levels of immune evasion driven positive selection. Similarly the N and C-heptad repeats need to productively interact with one another within the gp41 trimer [53] and the conserved residues in their coiled coil and helical domains required for these interactions [54] are understandably evolving under strong purifying selection.

**Figure 2:**
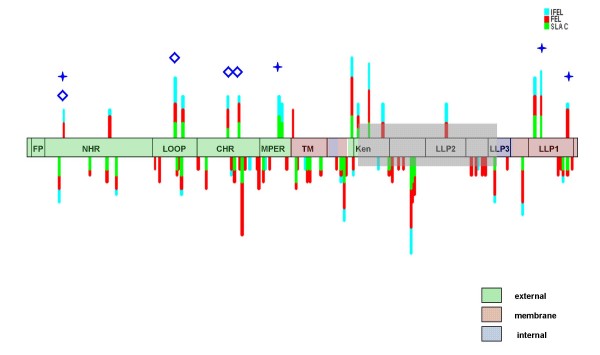
Graphical representation of the sites under selection seen in table 1 on a consensus scheme of the gp41 domains. Each detection method is shown in a different colour. Positively selected sites are at the top and negatively selected sites are on the bottom. The height of the top bars is proportional to the number of subtypes in which the position is detected as evolving under positive selection. On the underside only sites detectably under purifying selection in more than 3 datasets are represented. The diamonds denote sites detected to be evolving under positive selection by Travers et al (2005), while stars denote sites detected to be evolving under positive selection by Choisy et al (2003). The area overlapping the rev exon 2 is shaded in grey.

Seventeen gp41 sites were consistently detected to be evolving under positive selection in two or more of the nine analysed datasets (i.e. in at least two different subtypes or CRFs; Table 1 and Figure 2). All of these sites other than that at position 172 were also detectable evolving under positive selection by more of the three analysis methods. Of these 17 sites, five were situated in the overlapping *rev *exon 2 reading frame and, due to the confounding effects of overlapping reading frames on the inference of selection [55], these sites should probably be discounted. Nevertheless, the twelve other identified sites are presumably globally subject to the same selective pressures and might therefore indicate good targets for broadly effective treatment or vaccine interventions.

**Table 1:** The positions of sites identified as under positive selection across multiple HIV-1M lineages.

Codon position (HXB2 gp41)	Selection analysis method			Detected elsewhere^*a*^
		
	SLAC	FEL	IFEL	
24		B, D	B, D	T, C
54		B, F, CRF 02_AG	CRF 01_AE	
96	C, D	C, A, D	C, B, D, CRF 01_AE	T
101	B, G	B, G	B, G	
130	B	C, A, B	C, B	T
137	A, B	A, B, G	B	T
163	C, D, CRF 02_AG	C	C, D, CRF 02_AG	C
165	D, G		C, A, G	
172		C, B, G, CRF 02 _AG		
210	C, A, CRF 01_AE	C, A, B, D, F, G	C, D, CRF 01_AE	
214^*b*^	A	A, B	A, B	
221	G, CRF 01_AE	C, A, G, CRF 01_AE, CRF 02_AG	C, D, G, CRF 01_AE	
230		A, D	A, G, CRF 01_AE	
271		C, CRF 01_AE	C, B, D	
328	C, B, G	C, B, G	C, B, G	
332	A, B, G	A, B, G	B, D, G, CRF 01_AE	C
349		C, F, CRF 01_AE, CRF 02_AG	CRF 02_AG	C

^*a *^T = Travers *et al *(2005), C = Choisy *et al *(2003).

^*b*^Highlighted in yellow are sites that fall within the overlapping reading frame of the rev exon 2.

Studies by Choisy *et al*. [48] and Travers *et al*. [49] have used multiple subtypes to respectively identify nine and eight sites evolving under positive selection in gp41. Whereas the Choisy *et al*., study focused on comparing the locations and strengths of positive selection signals in different HIV-1 sequence alignments, that of Travers *et al*., focussed on likely selective pressures that have consistently shaped the evolution of HIV-1 group M *env *sequences since their diversification from the original group M founder virus. Choisy *et al *used a set of four subtype-specific alignments in their analysis and Travers *et al*., used a single alignment of 40 sequences containing viruses from multiple subtypes. Although both these studies used a set of maximum likelihood methods with six models of codon substitution, neither took recombination into account. Despite, the different methodologies and datasets used between our analysis and these two other studies, seven of the twelve sites we have identified as convincingly evolving under positive selection across multiple subtypes were also identified in these other studies. Importantly, our list helps reconcile differences between these other studies in that it includes six sites that were identified in one but not the other of the studies. This both confirms the robustness of the methodology we have employed and adds credibility to the notion that the five other sites we have identified have also probably been evolving under positive selection since the origin of the HIV-1 M subtypes.

The locations of both the 12 positively selected gp41 sites falling outside the overlapping *rev *exon and the five within the exon were examined in relation to probable glycosylation sites (PNGs), the position on the envelope spike, and the presence of CTL and nAb epitopes. Glycosylation in gp41 appears to be required for stabilisation of fusion active domains and efficient functioning [56] rather than for immune escape. We accordingly found no evidence of enrichment of positively selected codons associated with PNGs. We also found no significant association between the locations of CTL or nAb epitopes and sites under positive selection. We obtained the same results when all sites detected by two or more methods in each subtype were considered.

Given that the majority of nAb sites are in the external exposed domains of gp41, we analysed the sequences encoding these regions separately from the rest of the gene. In contrast with our previous result, within these domains alone, of the 173 sites analysed, the nine sites detected to be under positive selection in multiple datasets (Table 1) had a significant tendency to be located within neutralizing and other antibody epitopes (p = 0.01356: chi squared). The LLP1 domain alone has 3 sites evolving under positive selection, two of which were previously identified by Choisy *et al*. The LLP domains influence the surface expression of Env [57] and it is conceivable therefore that they may affect susceptibility to major broadly neutralizing antibodies such as 4E10 and 2F5 that target gp41.

### Differences in the selection signals detectable in sequences sampled during acute and chronic HIV infections

It is probable that the HIV transmission chain comprises a repetitive series of selective sweeps that intermittently remove much of the maladaptive evolutionary baggage that accumulates under host specific selection. Whereas this cyclical selection has probably amplified many of the positive selection signals detectable in the coding regions of HIV genomes, the strength and pervasiveness of these signals is obstructive when it comes to pinpointing when during the course of infection particular codons are evolving under positive selection. To identify sites evolving under different selection pressures at different times during infection, intuitively it might seem as though one need only sample some sequences during a particular infection phase and compare the selection signals detectable in these to the signals detected in sequences sampled during a different infection phase. The problem with doing this, however, is that inferring the types of selection operating on individual codons involves examination of the entire phylogenetic history of the sequences in question. Thus selection signals detectable in sequences sampled during acute infections may have been generated by selective process operating during the portion of their evolutionary histories spent in the chronic phases of past infections.

It is however possible that the cyclical purging of deleterious immune evasion mutations during acute and early infections coupled with the influence of a selective "transmission sieve" [14] might have left marks of selection on sequences sampled during acute infections that differentiated them from sequences sampled during chronic infections. We hypothesised that while viruses sampled during the acute phase of infection should carry slightly fewer signals of positive selection arising from transient maladaptive immune escape mutations, they might instead carry unique selection signals indicative of long-term adaptation that would otherwise be obscured in sequences sampled from chronically infected individuals.

**Figure 3:**
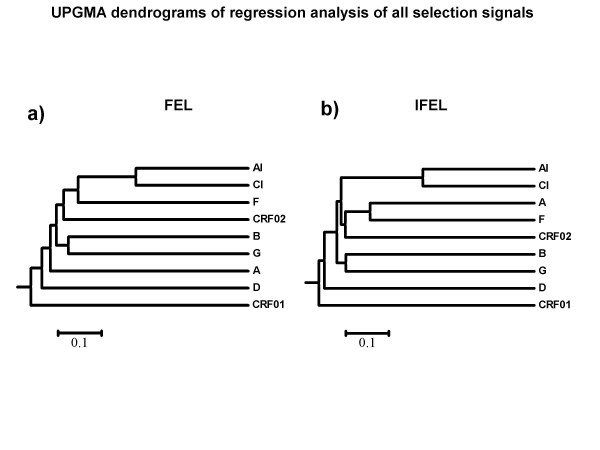
UPGMA dendrogram of regression coefficient matrices of normalised dN/dS ratios of every codon detected to be under any form of selection detected by FEL (a) and IFEL (b) methods across 9 different datasets.

To test this hypothesis we compared selection signals detectable by various methods in the subtype-C acute infection (AI) and chronic infection (CI) gp41 datasets in the context of selection signals detectable in datasets drawn from other HIV subtypes and circulating recombinant forms. We devised a simple linear regression test that could be used to visualise relationships between the selection signals detectable in different datasets. Given the largely overlapping evolutionary histories of the two subtype C datasets (Additional file 2), it was important that we determine whether they also shared selection signals that were more similar to one another than to those detectable in other HIV-1 subtypes. This test clearly indicated that selection signals detectable in the AI and CI subtype C datasets were more similar to one another than were any other pair of signals we compared (Figure 3a and 3b comparing signals detectable by the FEL and IFEL methods, respectively).

Given that the shared evolutionary histories of the AI and CI datasets are contributing to many of the selection signals detectable in both, we sought to determine whether certain subsets of codons within gp41 were detectably evolving under different selection pressures in the two datasets. To do this we partitioned all nine datasets into sites for which there was significant evidence (p < 0.05) of either positive or negative selection in any one of the nine gp41 datasets. These "positive" and "negative" datasets were further subdivided into three datasets each containing sites that, in any one of the nine datasets, were detectably evolving under positive or negative selection by (i) the FEL method, (ii) the IFEL method and (iii) the FEL method but not the IFEL method.

Whereas both the FEL and IFEL methods detect selection signals associated with the internal branches of phylogenetic trees, the FEL method also queries nucleotide substitutions that map to terminal tree branches and are thus assumed to have occurred more recently. According to our hypothesis, the most likely source of selection signals differentiating between our AI and CI datasets should be the substitutions occurring on these terminal branches. The reason for this is that, relative to the CI sequences, on average a greater proportion of the recent evolutionary histories of the AI sequences will have been spent in acute infections. By focusing on sites that were detectably evolving under positive or negative selection by the FEL method but not the IFEL method (i.e. sites in partition iii) we could test whether these selection signals were, as our hypothesis suggested they should be, less conserved between the AI and CI datasets than those detectable by the FEL and/or IFEL methods (i.e. sites in partitions i and ii).

**Figure 4:**
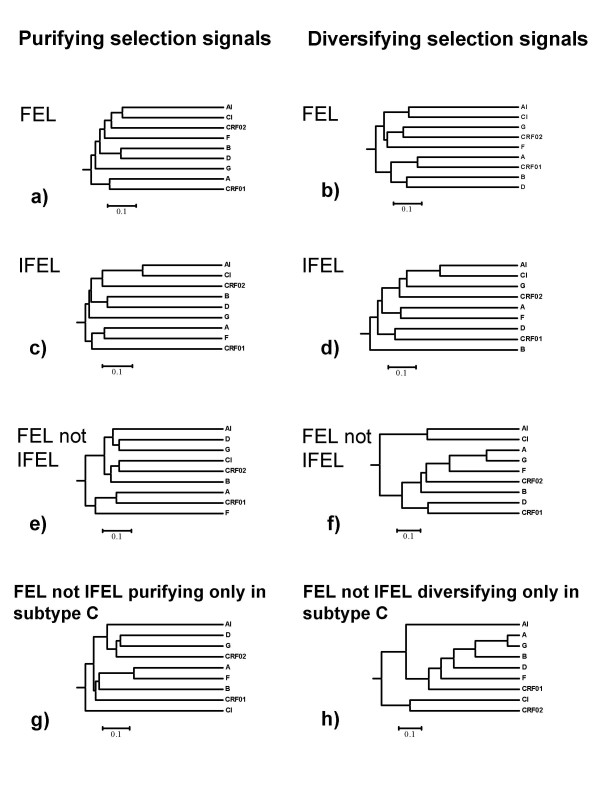
UPGMA dendrograms of regression matrices of normalised dN/dS ratios of codons detected to be under purifying (left) and diversifying (right) selection across 9 different datasets. Signals detected by FEL (a and b) IFEL (c and d) and FEL but not IFEL (e and f). In g and h, only sites detectably evolving under negative or positive selection by the FEL but not the IFEL methods in either one or both of these subtype C datasets are considered.

For both the negatively and positively selected site partitions examined with either the FEL or IFEL methods, the AI and CI datasets were more similar to one another than any other pair of datasets (Figure 4a to 4d). As we had anticipated, when only sites detectably evolving under positive selection by the FEL but not the IFEL method were considered, the AI and CI datasets were no longer the most similar two datasets examined (figure 4e and 4f). In the case of the negatively selected site partition the AI and CI datasets were no more similar to one another than either was to the other datasets examined. Importantly this relative decrease in the similarity of selection signals detectable in the AI and CI datasets is even more clearly evident when the only sites considered in the analysis are those detectably evolving under negative or positive selection by the FEL but not the IFEL methods in either one or both of these subtype C datasets (Figure 4g and 4h). This is consistent with our hypothesis that there are potentially acute and chronic infection associated selection signals within these datasets

It is important to point out that there was no significant difference between the AI and CI datasets with respect to the numbers of positive selection signals detected using the SLAC (8 in AI, 7 in CI), FEL (18 in both) and IFEL (12 in AI and 14 in CI) methods. As expected there were fewer signals detectable with the IFEL method than the FEL method because whereas the former only models selection along internal branches of the sequence phylogenies, the latter considers the entire tree.

### Selection signals differentiating acute and chronic infection datasets

Whereas no instances were found where there was statistically significant (P < 0.05) evidence of specific codons evolving under purifying selection in one dataset and under positive selection in the other using the FEL and IFEL methods, there were nevertheless 41 sites at which different selection pressures appeared to be operating in the two datasets. More than half of these (25) are sites where the differences between AI and CI were detected only by the FEL and not the IFEL method with the remainder either consistently different for both methods (6) or different for the IFEL method only (9).

From amongst the 31 sites where the FEL method indicated that there might be differences between selection signals in the AI and CI datasets, we focused on six sites where there was statistically significant evidence of selection in one direction in one dataset accompanied by selection in the other direction in the other dataset (Table 2).

**Table 2:** Codons evolving under different selection pressures in the AI and CI datasets.

Codon position (relative to HXB2 gp160)	Normalised dN/dS (SLAC, FEL, IFEL)^a^		Gp41 domain	nAb/CTL epitope
			
	AI	CI		
43 (554)	-1.62, **-0.36**, -0.37	0.50, 0.1, 0	N-heptad repeat	
48 (559)	-3.43, **-0.71**, -0.7	0.43, 0.1, 0	N-heptad repeat	RAIEAQQH- B, C & Cw
105 (616)^b^	-1.62, **-0.36**, -0.37	0.49, 0.03, 0	Loop region (glyc site)	
163 (674)^b^	-0.07, -0.04, 0.48	**2.88**, **0.19**, **0.44**	MPER	NWFNIT (4E10 nAb)
300 (804)^b^	0.22, 0.01, -0.04	**-1.62**, **-0.16**, -0.18	LLP3	LLQYWSQEL A*0201
309 (813)^b^	-0.24, -0.05, -0.04	2.14, **0.19**, 0.24	LLP3	QELKNSAVSL B60 & B*4001

^*a*^Significant (P < 0.05) values marked in bold typeface.

^*b*^sites at which selection signals were inferred to be significantly different between the AI and CI datasets using the PARRIS based test described in the methods.

Of these six sites, five are apparently evolving under purifying selection in the AI dataset but neutral or diversifying selection in the CI dataset. We specifically assessed these six sites for significant evidence of differential selection pressures using a test based on the relative effects likelihood based selection analysis method PARRIS [37]. This analysis provided additional evidence that four of these sites (105, 163, 300 and 309) were evolving under significantly different selection pressures in the CI and AI datasets (Table 2).

The two sites at which the PARRIS based test did not detect significant evidence of differential selection between the datasets were 43 and 48. According to the FEL method these sites appear to be evolving under strong purifying selection in the AI dataset but under either weak diversifying or neutral evolution in the CI dataset. They are within the N-terminal coiled-coil (NHR) region of gp41 and, interestingly, mutations at site 43 are associated with resistance to the HIV-1 fusion inhibitor enfuvirtide (fuzeon, or T-20) [58]. A 23 amino acid region of the N-heptad repeat containing these sites described by Moreno *et al *[59] interacts with negatively charged phospholipids initiating the conformational changes that result in disassembly of the envelope trimer core, fusion pore formation and six helix bundle formation that are essential for fusion. The essential function of this site presents a sound basis for it being subject to strong purifying selection. Whereas the PARRIS analysis confirmed that these sites were both evolving under strong purifying selection in the AI dataset, it also indicated that they were evolving under purifying selection (albeit apparently weaker) in the CI dataset.

Codon 105, a glycosylation site within the loop domain, is apparently evolving under strong purifying selection in the AI dataset but weakly positive or neutral selection in the CI dataset. In contrast with gp120, the gp41 ectodomain is relatively poorly glycosylated with only four or five potential glycosylation sites [60-62]. While these glycans do not detectably affect susceptibility to antibodies, their removal eliminates the ability of Env to mediate fusion [61]. Codon 105 is demonstrably the most functionally significant of all four glycosylation sites in gp41 as it is the only one capable of restoring fusion activity to envelopes with glycan free gp41 molecules [56].

Codons 163 and 309 are detectably evolving under strong positive selection in the CI dataset but under neutral or mildly negative selection in the AI dataset. Whereas site 163 is within the broadly recognized 4E10 neutralizing antibody epitope [63] and is understandably subjected to strong positive selection during chronic infections, site 309 is not within any well characterized CTL or antibody epitopes. The LLP domains within which site 309 is found may be directly exposed during fusion [64] and mutations here are also known to affect Env incorporation, virus infectivity and possibly virus exposure to neutralization [57]. Evidence of slightly purifying selection at sites 163 and 301 in the AI dataset may indicate that potential immune evasion mutations that occur at these sites during chronic HIV infections may incur "transmission fitness" costs.

Site 300 is the only site apparently evolving under significantly weaker negative selection in the AI dataset than is detectable in the CI dataset. Although it is not clear what the role of this site is in HIV-1 replication and pathogenesis, the apparent relaxation of selection at this codon during acute infection warrants further investigation.

## Conclusion

Although gp41 is the most conserved component of the envelope gene, it is evident that, as with the more variable gp120 encoding region, it contains a relatively large number of sites that are detectably evolving under positive selection. While we have compiled a map of conserved selection signals occurring in gp41 sequences of HIV-1M viruses, we have found interesting differences in the selection signals detectable in sequences sampled during acute and chronic subtype C infections. Our map of sites that have possibly been under consistent selection since the earliest HIV-1M ancestor and our discovery of selection signals distinguishing acute and chronic infections might help guide the development of broadly effective vaccine and treatment interventions. The gp41 gene may feature prominently in future vaccine strategies given that it contains many of the neutralizing epitopes identified to date.

The different selection signals we have detected in sequences sampled during acute and chronic infections might be rationally explained if one considers that viruses in acutely infected individuals may have a higher transmission frequency than viruses in chronically infected individuals [24]. One would expect that signals of selection should be clearest in sequences that are both sampled during acute infection and have moved along transmission chains in which they have spent a disproportionately large amount of time in acute infections. Although sequences sampled during chronic infections might have also experienced transmission chains with similar characteristics to those experienced by viruses sample during acute infections, they will have spent an average of a year or more prior to sampling within the evolutionary context of a chronic infection. This time will have been sufficient both for the reversion of slightly deleterious immune evasion mutations (and possibly their accessory compensatory mutations) that have occurred in former hosts [27,65,66] and the accumulation of novel mutations with adaptive value in their current hosts.

The selective sieve of transmission and the selective sweeps that presumably follow it are still poorly understood and might remain so unless genetic characteristics differentiating viruses sampled during acute and chronic infections are identified. Current evidence relating to the selective nature of the transmission bottleneck and acute infection remains somewhat contentious [31]. Our analysis reveals subtle differences in the distributions of sites evolving under positive and negative selection in chronic and acute subtype C infections. This implies that selective processes such as a transmission sieve might indeed be in operation – a possibility that is supported by the fact that some of the gp41 residues apparently evolving under stronger purifying selection during acute infection are involved in fusion or transmission related functions.

We have shown that across multiple HIV-1M subtypes and CRFs there are at least 12 gp41 sites that are detectably evolving under positive selection. While a vaccine that is protective against the transmitted viruses of particular HIV subtypes (or even smaller genetic clades within these subtypes) would be a major advance, the holy grail of vaccine research remains the development of an HIV vaccine that will protect against all HIV genetic variants. The fact that we and others have found, using a variety of inference tools, the same set of gp41 sites evolving under positive selection in a range of different HIV-1 subtypes indicates a degree of consistency in both the immunogenicity of these sites and the ways in which host immune systems are most likely targeting them. Given this consistency it may be possible to design a set of broadly protective vaccine immunogens that will induce simultaneous immunity to the common genetic variants found at these positively selected sites. While vaccines that induce immunity to the common genetic variants of these gp41 sites might be only partially protective, they should at the very least constrain the viruses' evolutionary options and, in so doing, potentially precipitate the evolution of decreased population-wide HIV pathogenicity.

We have shown that variations in the selective pressures experienced by viruses during the acute and chronic stages of infections might be both detectable by comparing sequences sampled during these infection phases, and a useful means of identifying viral genetic features that are important during either transmission or early infection. Identifying the key genetic determinants of HIV transmissibility through similar but more detailed analyses of selective forces associated with the transmission bottleneck and acute infection should not only identify good targets for treatment and preventative interventions but also inform the biochemical basis on which these interventions might operate.

## Methods

### Sequence datasets

Our acute infection dataset was derived from a subtype C acute infection study in Durban, South Africa (CAPRISA 002 Acute Infection cohort) that is currently following a cohort of HIV-negative high risk individuals and enrolling study participants upon seroconversion [67]. Long-template HIV-1 cDNA transcripts were generated from viral RNAs extracted from plasma from the first HIV sero-positive plasma sample of 40 study participants. The time of infection was defined as the mid-point between the last sero-negative and first sero-positive visits. Given this estimate plasma samples were on average obtained at 40 days post infection. Whole genomes were amplified from cDNA using a modified limiting dilution nested PCR assay as described by Rousseau *et al *[68]. First-round whole-genome products were used as templates to amplify full-length envelope genes. The ~3-kb PCR fragments which included the entire gp160 were amplified using the previously described envA and envM primers [69] and directly sequenced. The gp41 region of these 40 sequences represented an acute infection dataset (AI dataset) which we used to identify genetic features characteristic of early infection [All Genbank: FJ229799, FJ229800, FJ229801, FJ229802, FJ229803, FJ229804, FJ229805, FJ229806, FJ229810, FJ229808, FJ229809, FJ229807, FJ229811, FJ229812, FJ229813, FJ229814, FJ229815, FJ229816, FJ229817, FJ229818, FJ229819, FJ229820, FJ229821, FJ229822, FJ229823, FJ229824, FJ229825, FJ229826, FJ229827, FJ229828, FJ229829, FJ229830, FJ229831, FJ229832, FJ229833, FJ229834, FJ229835, FJ229838, FJ229837, FJ229836]

A chronic infection dataset (CI dataset) consisting of 40 gp41 subtype C sequences, was derived from a previous study conducted in Durban [70] [All Genbank: AY463221, AY463222, AY463230, AY463232, AY463233, AY463234, AY772699, AY838566, AY838567, AY878055, AY878059, AY878061, AY901977, DQ011165, DQ275648, DQ275652, DQ275658, DQ351229, DQ351235, DQ369989, DQ396378, DQ396380, AY463219, AY463226, AY463231, AY463236, AY463237, AY703908, AY703911, AY772690, AY772691, AY772696, AY878054, AY878056, AY901969, AY901965, AY901968, AY878072]. These sequences were generated from subjects with established infections that matched the AI dataset for geographical location, race and gender. To minimize artifactual noise due to AIDS defining illness, sequences from participants with CD4+ counts <200 cells/μl were excluded. In addition, sequences were also excluded if viral loads were > 200 000 copies/ml.

Non-subtype C gp41 nucleotide sequence alignments were obtained from the Los Alamos National Laboratory HIV sequence database (LANL dataset; ) as follows; subtype A (n = 40) [All Genbank; DQ396400, AB253428, AF004885, U08794, AF069670, AF069671, AF407148, AF407151, AF286237, L22957, AF361872, AF484507, AF484478, AF457052, AF457055, AF457063, AF457066, AF457068, AF457077, AF286241, AF286238, AF413987, Y13718, Y13717, L22951, L07082, AY829203, AY829205, AY829206, AJ401040, AM000053, AM000054, AF219265, DQ167216, DQ207944, DQ083238, DQ823358, EF589039, AM279348, AY521629], subtype B (n = 40) [All Genbank; U08441, U08443, U08444, U08446, U08447, U23487, U08445, AF112539, AF490512, AY037270, AY037269, AY037282, AJ417411, AJ417420, AJ417429, AF041125, AF041132, AF041134, AF277054, AY308760, DQ295193, AY314044, AF277055, AF277056, AF277058, AF277074, AF538303, AY561236, AY561237, AY561239, AY751406, AY835447, AY835449, DQ085869, DQ085870, AB262952, AF277061, AF277064, AF277065, AF277063], subtype D (n = 40) [All Genbank; J03653, U36884, U36887, AJ277820, AF321082, AF219272, DQ141204, A34828, U27399, U27419, U08805, AY494966, U88822, A07108, K03458, AF133821, AY669758, AY713418, AF484504, DQ054367, AJ488926, AF484499, AF457090, U65075, U36867, AY253311, AY322189, AY773340, AY773341, AY371157, AY371156, AY371155, AJ401037, AF484502, AY795907, AY304496, L22945, L22950, L22949, AY795903], subtype F (n = 28) [Genbank; AF005494, AF075703, AF077336, AF377956, AJ249236, AJ249237, AJ249238, AJ277819, AJ277824, AY173957, AY173958, AY231157, AY371158, AF005494, DQ189088, DQ313239, DQ313240, DQ313241, U27401, DQ979023, DQ979024, DQ979025, EF374130, EF374131, L22082, L22085, DQ358801], subtype G (n = 34) [Genbank; U09664, AF069935, AF069937, AF069943, AF069947, AY772535, AY586547, AY586548, AY586549, AY612637, EF025323, U27426, U27445, U88826, AF061642, AF084936, AF450098, AF423760, AY371121, AY231155, AY231156, AF061640, DQ168573, AM279346, DQ168576, DQ168579, EF033659, AB231893, EF367208, AM279365, AM279351, AM279359, AM279350, DQ168575], CRF 01_AE (n = 36) [Genbank; U08456, U08457, U08458, U51188, AF070703, AF070704, AF070709, AF070710, AF070711, AF070712, U09131, U39256, AB070352, AB052995, AY494967, AB032740, AB032741, AY231158, AF219273, AY444803, AY444804, AY444805, AY444806, DQ859178, DQ859179, EF036536, DQ354117, EF036527, EF036529, EF036530, EF036531, EF036532, EF036533, EF036534, EF036535, DQ859180] and CRF02_AG (n = 40) [Genbank; AF069933, AF069941, AF107770, AF321079, AB049811, AY271690, DQ313247, AB231898, AB231896, AB231895, AB231894, AF063223, DD409979, AF377954, AF377955, L22939, AY151001, AY151002, AY231152, AY231153, AY829204, AY829207, AY829214, AF063224, AJ251057, AJ251056, AY371125, AY371126, AY371127, AY371128, AY371129, AY371130, AY371131, AY371132, AM279360, AY371139, AY371140, AY371146, AY736840, AY371137].

All datsets were aligned using the ClustalW method [71]. These alignments were then edited by eye and are available from the authors on request.

### Recombination analysis

Recombination breakpoints were detected in all datasets using the GARD method [40] implemented on the datamonkey web server  Analyses were run using the HKY85 nucleotide substitution model with no rate variation (determined automatically to be the best model by a built in model selection procedure) with the transition:transversion ratio being determined from the data.

The distribution of unambiguously detected breakpoint positions of all unique recombination events detectable in a composite of all analysed gp41 datasets, (excluding the AI subtype C dataset to ensure that individual recombination events were counted only once) were analysed for evidence of recombination hot- and cold-spots with RDP3 [41] as described previously [72]. Briefly this involved for each individual dataset, detection of recombination breakpoints using the RDP [73], GENECONV [74], BOOTSCAN [46], MAXCHI [75], CHIMAERA [76], SISCAN [77], and 3SEQ [78] methods implemented in RDP3. Default settings were used throughout and only potential recombination events detected by two or more of the above methods, coupled with phylogenetic evidence of recombination were considered significant. Using the approach outlined in the RDP3 program manual (available from , the approximate breakpoint positions and recombinant sequence(s) inferred for every potential recombination event were manually checked and adjusted where necessary using the extensive phylogenetic and recombination signal analysis features available in RDP3.

### Selection analysis

Breakpoint positions inferred in the GARD analyses were fed directly into the single likelihood ancestor counting (SLAC) [79] fixed effects likelihood (FEL) [80] and internal fixed effects likelihood (IFEL) [81] analyses implemented on the Datamonkey web server for site-by-site identification of positively selected codons. The methods are implemented as a series of HyPhy scripts [80] that yield site-specific evidence of positive and purifying selection. Using the results from the GARD screen the methods make allowance for both independent inference of phylogenetic parameters over tracts of sequence separated by recombination breakpoints and variable synonymous substitution rates across gp41.

SLAC is a counting method that, given a set of input sequences, an associated phylogeny and a codon based substitution model, involves counting the number of synonymous and non-synonymous changes that occur at a given site. This method examines nucleotide substitutions inferred to have occurred on every branch of the phylogeny and incorporates weighting of nucleotide substitution biases estimated from the data to determine whether more or fewer non-synonymous substitutions have occurred at particular sites than would be expected by chance.

Whereas the FEL method also uses the phylogenetic context of all nucleotide substitutions in the history of a sample of sequences to detect evidence of positive and negative selection, the IFEL method searches for selection signals only amongst those nucleotide substitutions that have apparently occurred within the shared evolutionary histories of at least two different sequences in an analysed sample – i.e. it only counts substitutions that can be mapped to the internal branches of an associated phylogenetic tree. Using FEL and IFEL together allows one to phylogenetically distinguish between sites where signals of selection are mainly detectable amongst nucleotide substitutions associated with the terminal branches of phylogenetic trees – such as, for example, mutations that are specifically adaptative in individual hosts – and selection that occurs along internal tree branches – such as, for example, selection associated with ancestral adaptations in global populations [81]. Details of the methods and HyPhy scripts used are available on the Datamonkey website.

We compared signals of selection between all pairs of HIV gp41 datasets using linear Pearson regression analysis of normalised dN/dS ratios of either all or selected subsets of individual codons along the length on the gene. For each pair of gp41 datasets one minus the correlation coefficient, R, was used as a measure of their similarity. UPGMA [82] and neighbour joining [83] clustering algorithms were then used to visualise how closely the selection signals in different datasets (represented by a matrix of 1-R values) resembled one-another.

To obtain additional statistical data on differential selection signals detectable in our AI and CI datasets, we used the relative effects likelihood based PARRIS selection analysis method. This method uses likelihoods ratio tests to fit each codon to one of 3 models of selection (purifying, neutral and positive) and assigns a posterior probability for each rate class at each site. As with the SLAC, FEL and IFEL methods PARRIS also takes recombination and synonymous rate variation into account [37].

We devised a simple statistical test to determine whether homologous sites in the two different datasets were evolving under significantly different selection pressures. We used PARRIS to estimate posterior probability distributions of ω for each site and from these we estimated the mean and variance of ω. A variable synonymous substitution rate was selected, and the M1a and M2a models [84] were compared to detect positive selection. We calculated the mean estimated ώ value (or μ) for each site using the formula μ = ∑ P_C _ώ_C_, where P_C _is the posterior probability for each selection class. The standard deviation (SD) of each estimate of μ was calculated as SD = (∑ P_C_(μ-ώ_C_)^2^)^0.5^. The estimated value of ω ± standard deviation at each site was calculated and sites at which these ranges did not overlap were considered to have signals of significantly different selection pressures between the datasets.

### RNA secondary structure prediction

RNA secondary structure predictions were carried out using the alignment based RNA folding tool, GeneBee available online at [85] -. Default settings were used throughout.

### Glycosylation analysis

Detection of putative N-linked Glycosylation (PNGS) sites was carried out using the online tool, N-Glycosite, available at [86]. Default settings were used.

### Epitope Mapping

Maps of CTL/CD8+ and neutralizing and binding antibody epitopes were obtained from the Los Alomos HIV Molecular Immunology database which is publicly available at 

### Statistical tests

GraphPad Prism (version 4; GraphPad Software) was used for statistical analyses. Tests of association between neutralizing antibody and CTL/CD8+ epitopes and the presence of positively selected sites were carried out using 2-tailed Fisher's exact tests.

## Competing interests

The authors declare that they have no competing interests.

## Authors' contributions

GB carried out the molecular genetic studies, participated in PCR amplification, sequence alignment, data analysis and drafting of the manuscript. FT helped with PCR amplification on many of the sequences. DM is the corresponding author. He assisted in the conception of data analyses, and in writing the manuscript. KM is the project director of the CAPRISA AI 002 Study. SSAK is the director of CAPRISA. CW is the supervising author.

CAPRISA AI Team are responsible for running the AI 002 study. They interfaced with participants and collected all AI samples used in this work.

All authors read and approved the final manuscript

## Supplementary Material

Additional file 1 Combined intra-subtype recombination breakpoint distributions detectable within eight subtype and circulating recombinant for gp41encoding nucleotide sequence datasets. Whereas the broken lines denote 99% and 95% confidence intervals for Heath's global breakpoint clustering test [72], the light and grey regions respectively denote 99% and 95% confidence intervals for Heath's local breakpoint clustering test. A map of gp41 domains is given for orientation purposes. Whereas green regions represent portions of the encoded protein found exposed external surfaces of viral particles, red regions represent membrane embedded domains and blue regions domains that are within the virus particle. Click here for file

Additional file 2 Neighbour joining tree of 40 CAPRISA AI and 40 CI gp41 sequences. This tree demonstrates that the AI and CI subtype C datasets do not originate from separate phylogenies and have largely overlapping evolutionary histories Click here for file
