# Supplementary material for: Conserved positive selection signals in gp41 across multiple subtypes and difference in selection signals detectable in gp41 sequences sampled during acute and chronic HIV-1 subtype C infection
Source: Virol J. 2008 Nov 24;5:141. doi: 10.1186/1743-422X-5-141 (PMC2630941; doi:10.1186/1743-422X-5-141)
Supplement: Additional file 1 — Combined intra-subtype recombination breakpoint distributions detectable within eight subtype and circulating recombinant for gp41encoding nucleotide sequence datasets. Whereas the broken lines denote 99% and 95% confidence intervals for Heath's global breakpoint clustering test [72], the light and grey regions respectively denote 99% and 95% confidence intervals for Heath's local breakpoint clustering test. A map of gp41 domains is given for orientation purposes. Whereas green regions represent portions of the encoded protein found exposed external surfaces of viral particles, red regions represent membrane embedded domains and blue regions domains that are within the virus particle. [file 1743-422X-5-141-S1.ppt]

## Slide 1
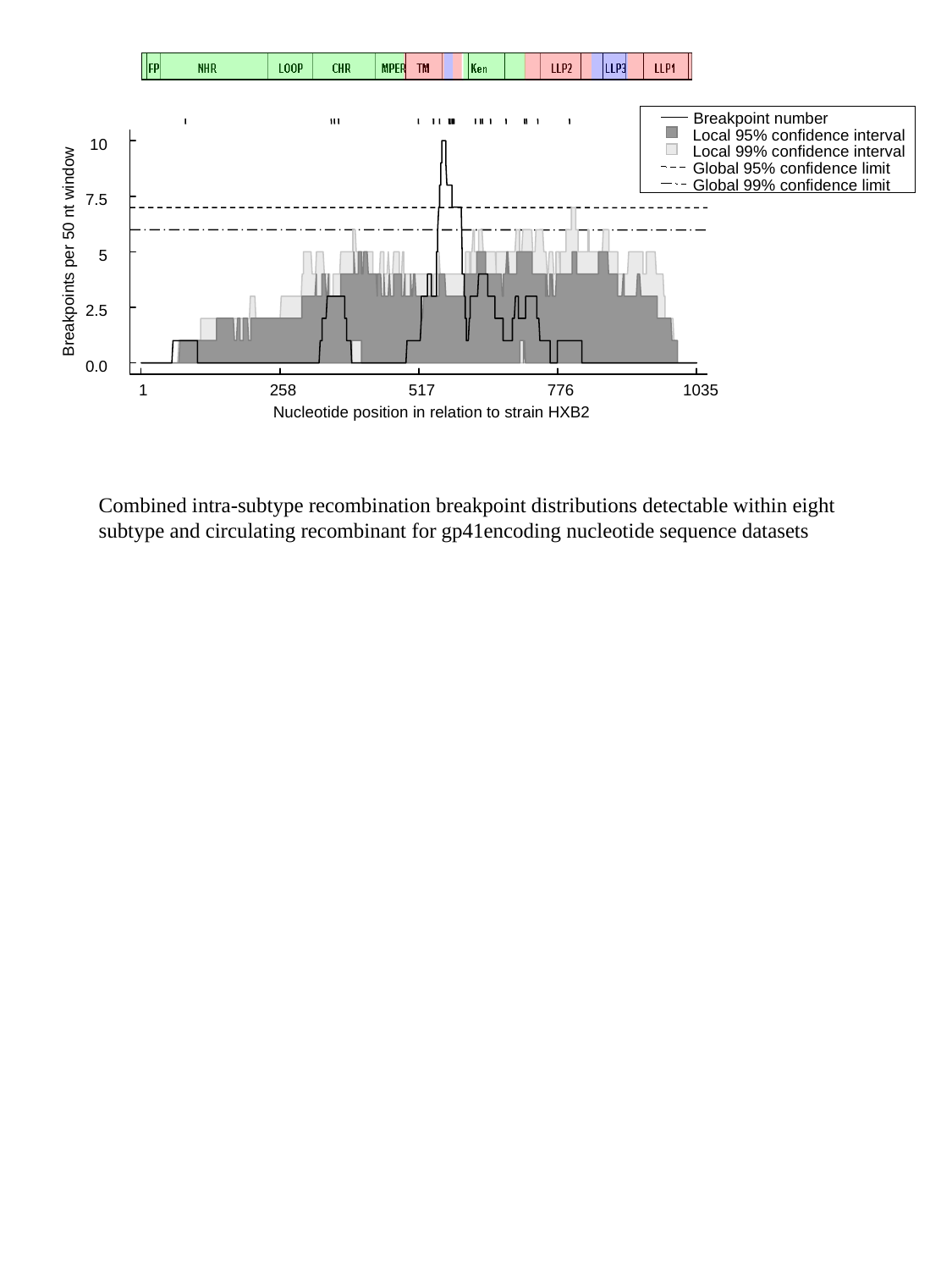

Breakpoint number
Local 95% confidence interval
10
7.5
Breakpoints per 50 nt window
5
2.5
0.0
1
258
517
776
1035
Nucleotide position in relation to strain HXB2
Local 99% confidence interval
Global 95% confidence limit
Global 99% confidence limit
Combined intra-subtype recombination breakpoint distributions detectable within eight subtype and circulating recombinant for gp41encoding nucleotide sequence datasets
