# Supplementary figures and images for: Conserved positive selection signals in gp41 across multiple subtypes and difference in selection signals detectable in gp41 sequences sampled during acute and chronic HIV-1 subtype C infection
Source: Virol J. 2008 Nov 24;5:141. doi: 10.1186/1743-422X-5-141 (PMC2630941; doi:10.1186/1743-422X-5-141)

## Slide 1
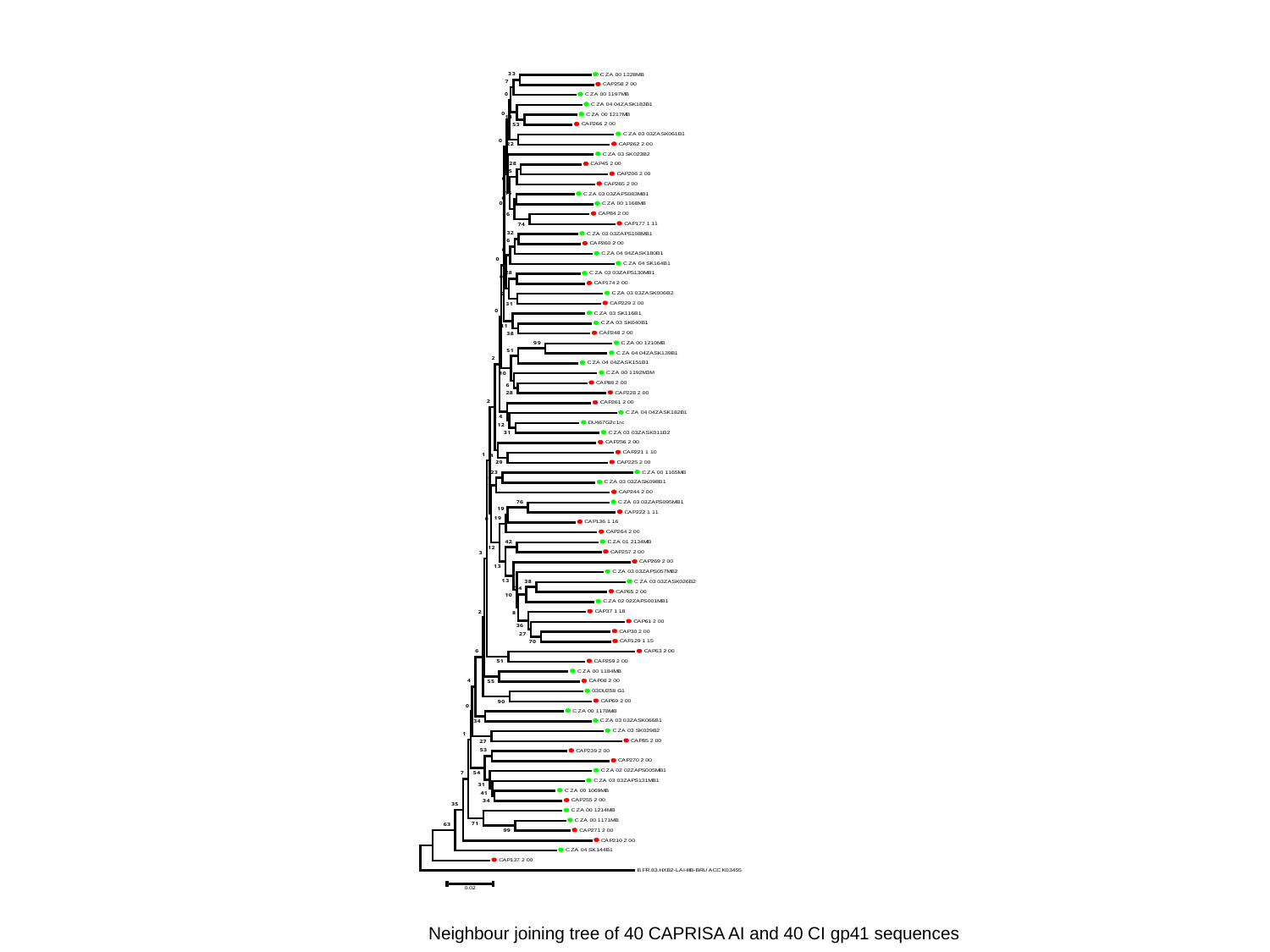

Neighbour joining tree of 40 CAPRISA AI and 40 CI gp41 sequences

Supplement: Additional file 2 — Neighbour joining tree of 40 CAPRISA AI and 40 CI gp41 sequences. This tree demonstrates that the AI and CI subtype C datasets do not originate from separate phylogenies and have largely overlapping evolutionary histories [file 1743-422X-5-141-S2.ppt]
